# Type material of South American *Pimpla* Fabricius, 1804 (Hymenoptera, Ichneumonidae) housed in the Instituto Fundación Miguel Lillo, Argentina

**DOI:** 10.3897/zookeys.1281.194880

**Published:** 2026-06-03

**Authors:** Diego G. Pádua, Emilia C. Pérez, Rodrigo O. Araujo

**Affiliations:** 1 Laboratorio de Entomología General y Aplicada, Centro de Investigación de Estudios Avanzados del Maule, Vicerrectoría de Investigación y Postgrado, Universidad Católica del Maule, Av. San Miguel 3605, Talca, Chile Instituto de Entomología, Fundación Miguel Lillo San Miguel de Tucumán Argentina; 2 Instituto de Entomología, Fundación Miguel Lillo, Miguel Lillo 251 (4000), San Miguel de Tucumán, Argentina Vicerrectoría de Investigación y Postgrado, Universidad Católica del Maule Talca Chile

**Keywords:** Darwin wasps, parasitoid wasp, Pimplini, taxonomy

## Abstract

In this study, we conducted a taxonomic examination of type specimens of South American *Pimpla* (Ichneumonidae) described by Charles Porter and deposited in the entomological collection of the Instituto Fundación Miguel Lillo (IFML), Argentina. This study aims to facilitate the morphological understanding of South American *Pimpla* species through the application of modern imaging techniques. A total of 11 species were examined: *P. aeola*, *P. alnorum*, *P. golbachi*, *P. jakulicai*, *P. oropha*, *P. pyramis*, *P. ramirezi*, *P. sparsa*, *P. stangei*, *P. tafiae*, and *P. tarapacae*. For all species, except *P. golbachi*, diagnoses distinguishing Porter’s species from other Neotropical species of *Pimpla*, together with digital images, are presented for the first time. In addition, a taxonomic key to females of the Argentine species of *Pimpla* is provided.

## Introduction

*Pimpla* Fabricius, 1804 (Ichneumonidae) is one of the largest genera in the subfamily Pimplinae, comprising more than 200 valid species worldwide (Yu et al. 2016; Watanabe and Matsumoto 2019; Pádua et al. 2020). Most species of the genus are idiobiont endoparasitoids, primarily attacking prepupae or pupae of Lepidoptera (Gauld 1991; Yu et al. 2016).

*Pimpla* is relatively well known in the Neotropical region due to taxonomic studies conducted in Central America (Gauld 1991; Gauld et al. 1998, 2002; Khalaim and Ruíz-Cancino 2021) and South America (Porter 1970; Díaz 2000; Pádua et al. 2019, 2020; Villanueva-Bonilla et al. 2021), and species of the genus are well represented in many entomological collections (Porter 1970).

In South America, Porter (1970) produced the only comprehensive taxonomic revision of the genus, recognizing 35 species, of which 21 were described as new. In that work, he also proposed species groups based on morphological characters and provided an identification key for the South American species. Later, Porter (1972) expanded this taxonomic framework by describing a new species, *Pimpla jakulicai*.

Despite the importance of these contributions to the knowledge of the genus in the region, the earlier studies were produced at a time when digital imaging was not commonly available and therefore relied mainly on schematic drawings of structures and appendages. Although these illustrations were valuable for their time, the absence of detailed digital images may now limit the accurate interpretation of some morphological characters and make the reliable identification of specimens more difficult. Furthermore, although Porter (1970, 1972) provided diagnostic features for the species he described, these diagnoses were mainly focused on comparisons with closely related taxa, limiting their applicability for distinguishing these species from all other valid Neotropical species of *Pimpla*.

Accordingly, the present study aims to provide diagnoses and digital images of the 11 *Pimpla* species represented by type material treated in Porter’s (1970, 1972) works and currently deposited in the Instituto Fundación Miguel Lillo (IFML). These species represent all the *Pimpla* type material from Porter’s studies available in this collection. By documenting these specimens, we aim to facilitate the understanding of external morphology and improve the accurate identification of some South American species. In addition, a taxonomic key for Argentine species is presented.

## Materials and methods

All examined specimens are deposited in the entomological collection of the Instituto Fundación Miguel Lillo, San Miguel de Tucumán, Argentina. Terminology and measurements follow Broad et al. (2018). The section “Material examined” includes the details provided on the label. Digital images were obtained using a Leica M205 C stereomicroscope fitted with the Leica DMC 2900 digital camera. All photos were edited in Adobe Photoshop 2020.

Label data are transcribed verbatim; information added by the authors is given in square brackets.

The abbreviations adopted in this study are as follows: **IFML** = Instituto Fundación Miguel Lillo; **EMUS** = Utah State University insect collection.

## Results

### Key to females of the Argentine species of *Pimpla* Fabricius, 1804

Taxonomic key adapted from Porter (1970). The female of *P. alnorum* is unknown.

**Table:** 

1	Fore wing hyaline to yellowish, with a large, dark, subapical/apical spot strongly contrasting; mesosoma yellow to orange-brown; mandible with a translucent basal flange; pleural carina of propodeum reduced or nearly absent; mesoscutum with three dark longitudinal stripes; metasomal tergites with dark transverse bands; hind coxa often with a dark dorsal stripe; basal face of propodeum with transverse wrinkles	***P. sumichrasti* Cresson, 1874**
–	Fore wing without a large strongly contrasting apical spot; mesosoma black, metallic, red, or with white/yellowish markings; mandible without a translucent basal flange; pleural carina of propodeum usually detectable	**2**
2	Metasoma predominantly orange; hind coxa orange; subalarum strongly swollen/callose, with a short carina behind; sides of propodeum with long, dense, silvery setae	***P. croceiventris* (Cresson, 1868)**
–	Metasoma not predominantly orange in this combination	**3**
3	Mesosoma with conspicuous white markings on pronotum, scutellum/postscutellum, posterior corners of propodeum, and/or mesopleuron; metasoma often with white apical bands	**4**
–	Extensive white markings absent or not arranged in this pattern; metasoma black, metallic, red, or brown, without a dominant pattern of broad white bands	**7**
4	Ovipositor sheath very short (Fig. 7A); sheathed portion about < 0.5 of hind tibia length (Fig. 7D); tergite II relatively long, about 0.87 as long as wide (Fig. 7E); subalarum dark (Fig. 7A); hind coxa without a large white spot (Fig. 7A)	***P. ramirezi* (Porter, 1970)**
–	Ovipositor sheath > 0.6 of hind tibia length; pattern of legs, tegula, and white markings different	**5**
5	Tegula largely white (Fig. 4A); metasoma black with white bands and extensive reddish colouration apically (Fig. 4A, E); fore femur reddish (Fig. 4A); fore tibia pale red; hind coxa red, only slightly darkened apically (Fig. 4A); propodeum less elongate, with stronger and more regular transverse rugosity	***P. jakulicai* (Porter, 1972)**
–	Tegula not predominantly white, or combination above absent	**6**
6	Subalarum largely white (Fig. 10A); posteroinferior corner of mesopleuron with a large white spot (Fig. 10A); hind coxa with a large dorsolateral white spot near base (Fig. 10A); fore tibia distinctly swollen (Fig. 10A); ovipositor sheath about 0.9 of hind tibia length (Fig. 10A, E)	***P. tafiae* (Porter, 1970)**
–	Subalarum and posterior corner of mesopleuron without such a large white spot (Fig. 9A); scape black with white on apical part (Fig. 9C); mesopleural punctation more regularly subadjacent; metapleuron with small to medium punctures and fine oblique rugulation	***P. stangei* (Porter, 1970)**
7	Body metallic blue or blue-green; wings hyaline to moderately darkened, sometimes with metallic reflections; lateral carinae of scutellum detectable along about 1/4–1/2 or more of its length	***P. caerulea* Brullé, 1846**
–	Body not metallic blue; mesosoma black/yellowish/orange or with another pattern	**8**
8	Mesosoma often with yellow markings; wings hyaline to yellowish; mid and hind tibiae and tarsi yellow	***P. flavipennis* (Enderlein, 1919)**
–	Mid and hind tibiae and tarsi not uniformly yellow, or metasoma with a black-yellow pattern and reddish-brown markings	**9**
9	Hind femur reddish brown with apical darkening, or almost entirely yellow; metasoma black and yellow, often with extensive reddish-brown colouration beyond tergites III–IV; punctation of mesoscutum mostly adjacent to confluent	***P. tomyris* Schrottky, 1902**
–	Without this combination; laterotergites and metasoma as below	**10**
10	Laterotergite II elongate and narrow, usually > 3 × as long as wide; laterotergite III somewhat wider, and those of tergites IV–V contrastingly shorter and broader; postpetiole and tergite II strongly punctate	**11**
–	Laterotergite II broader, short-wedge-shaped, about 1.3–2.6 × as long as wide; following laterotergites similar, not so strongly contrasting	**13**
11	Metasoma mostly black; wings strongly darkened, with metallic/purplish reflections; coxae shiny black; fore tibia only slightly swollen	***P. cyanipennis* Brullé, 1846**
–	Metasoma partly or entirely red, or black with brownish/whitish apical margins	**12**
12	Metasoma usually solid red (Fig. 3A, E); propodeum and metapleuron almost entirely black (Fig. 3A); hind tibia dull red, at most weakly darkened apically (Fig. 3A)	***P. golbachi* (Porter, 1970)**
–	Metasoma red basally but darkening toward apex; propodeum and metapleuron partly to almost entirely red; hind tibia red with apical half, or more, distinctly black	***P. semirufa* Brullé, 1846**
13	Mid and hind coxae black; tegula without white; metasoma black with brown or whitish apical margins	***P. fuscipes* Brullé, 1846**
–	Mid and hind coxae not black in this combination; hind tibia and trochantelli orange or mostly orange	**14**
14	Hind tibia uniformly orange (Fig. 5A); facial punctation more widely spaced below and laterally (Fig. 5C); temple about 1.0 × eye length (Fig. 5A, C); mesoscutum very sparsely punctate except on basal third; scutellum broad and flat; postpetiole short, about 0.61–0.63 as long as wide	***P. oropha* (Porter, 1970)**
–	Hind tibia not uniformly orange; mid and hind trochantelli predominantly orange; lower half of mesopleuron with subadjacent to adjacent or reticulately confluent punctation, often with intercalated longitudinal rugulation; metapleuron with many strong, medium-sized, adjacent to confluent punctures	***P. patirrufa* Pádua et al., 2020 (= *P. rufipes* Brullé, 1846; see Pádua et al. 2020)**

### Taxonomy

#### Pimpla

Taxon classificationAnimaliaHymenopteraIchneumonidae

Fabricius, 1804

2FD49399-8080-5B15-A185-0B6B3EE17024

Pimpla Fabricius, 1804: 112. Type species: Ichneumon instigator Fabricius, 1793 (= Ichneumon hypochondriaca Retzius, 1783), by subsequent designation (Opinion 159, International Commission on Zoological Nomenclature 1945: 282).

##### Generic synonymy.

The generic concept of *Pimpla* adopted here follows the broad circumscription currently used for Pimplini, in which *Coccygomimus* Saussure, 1892, *Habropimpla* Cameron, 1900, *Lissotheronia* Cameron, 1905, *Phytodiaetoides* Morley, 1913, *Pimplidea* Viereck, 1914, *Coelopimpla* Brèthes, 1916, *Liotheronia* Enderlein, 1919, *Dihyboplax* Enderlein, 1919, *Neogabunia* Brèthes, 1927, *Opodactyla* Seyrig, 1932, *Oxypimpla* Noskiewicz & Chudoba, 1951, and *Jamaicapimpla* Mason, 1975 are treated as junior synonyms of *Pimpla*. For full nomenclatural details, including type-species designations, see Gauld (1991) and Pádua et al. (2020).

##### Diagnosis.

*Pimpla* can be distinguished from other genera by the following combination of characters: inner orbital margin weakly to distinctly concave above the antennal socket; clypeus entire, not divided by a transverse suture; malar space 0.35–1.4 × as long as the basal width of the mandible; mandible broad, with the upper tooth approximately subequal in length to the lower tooth; notauli weakly developed or absent, without a distinct anterior crest; propodeum with median longitudinal carinae absent to weakly traceable, and pleural carina usually present, although occasionally reduced or absent; fore wing 2.7–18.0 mm long; hind femur without ventral teeth; tarsal claws large and simple, lacking a basal lobe and without an enlarged flattened apical hair; metasoma densely punctate to nearly impunctate; and, in females, ovipositor nearly straight, with the apex never sharply decurved (Porter 1970).

#### Pimpla aeola

Taxon classificationAnimaliaHymenopteraIchneumonidae

(Porter, 1970)

977A84B1-6FE4-58DF-91F7-D9762157EBC8

Fig. 1

Coccygomimus aeolus Porter, 1970: 136. Holotype female, Peru (EMUS).

##### Diagnosis.

**Figure 1.**
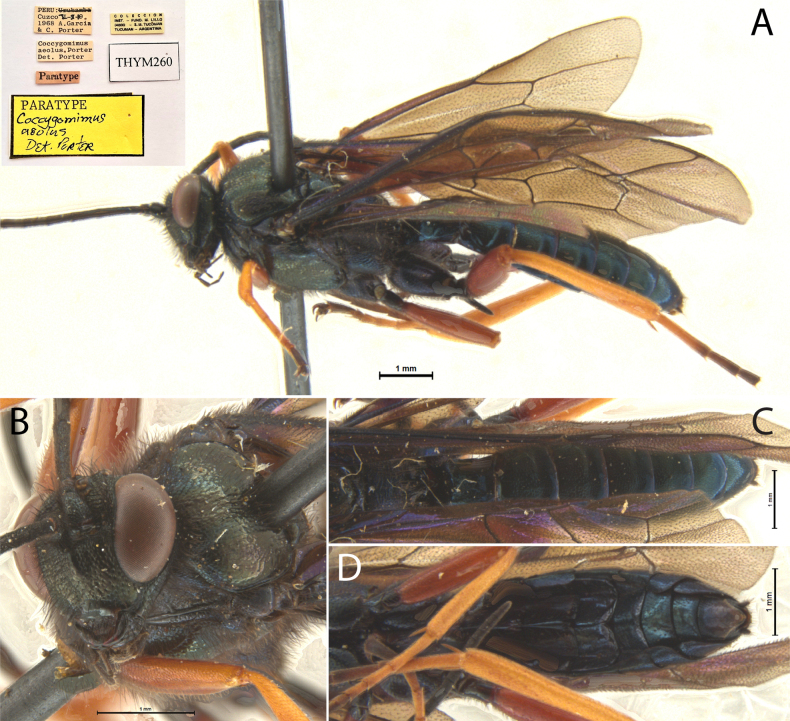
*Pimpla aeola* (Porter, 1970), male, paratype. **A**. Body, lateral view; **B**. Face, frontal view; **C**. Metasoma, dorsal view; **D**. Metasoma, ventral view.

**Figure 2.**
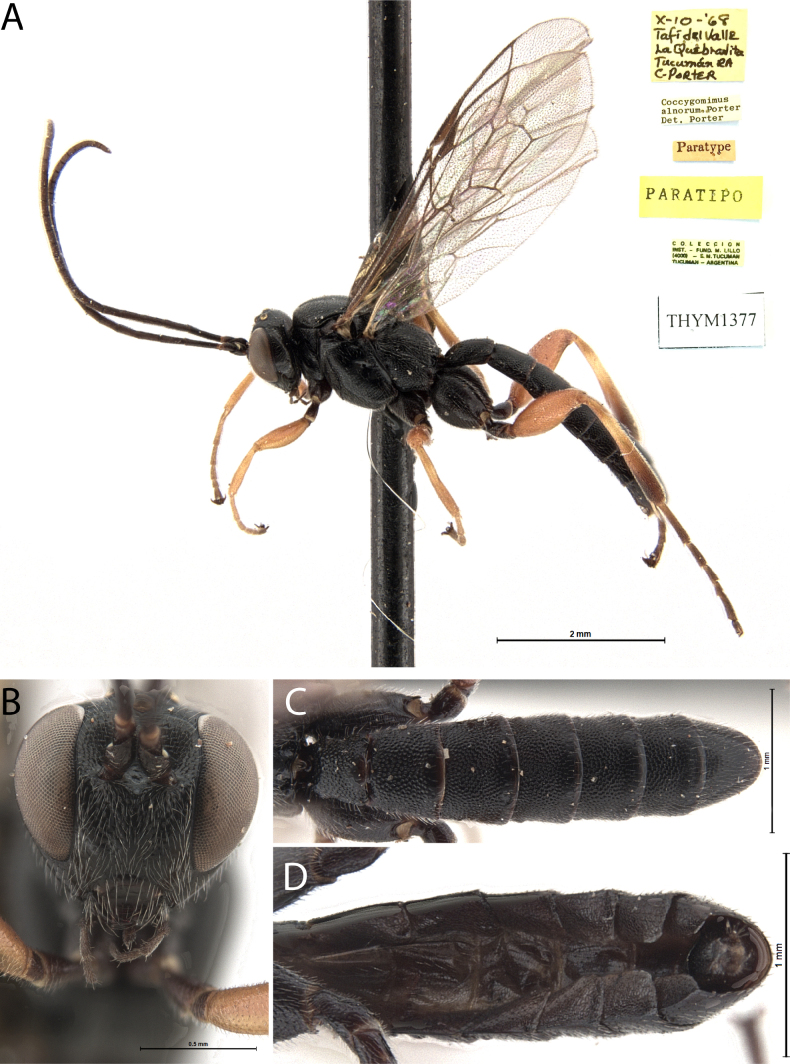
*Pimpla alnorum* (Porter, 1970), male, paratype. **A**. Body, lateral view; **B**. Face, frontal view; **C**. Metasoma, dorsal view; **D**. Metasoma, ventral view.

**Figure 3.**
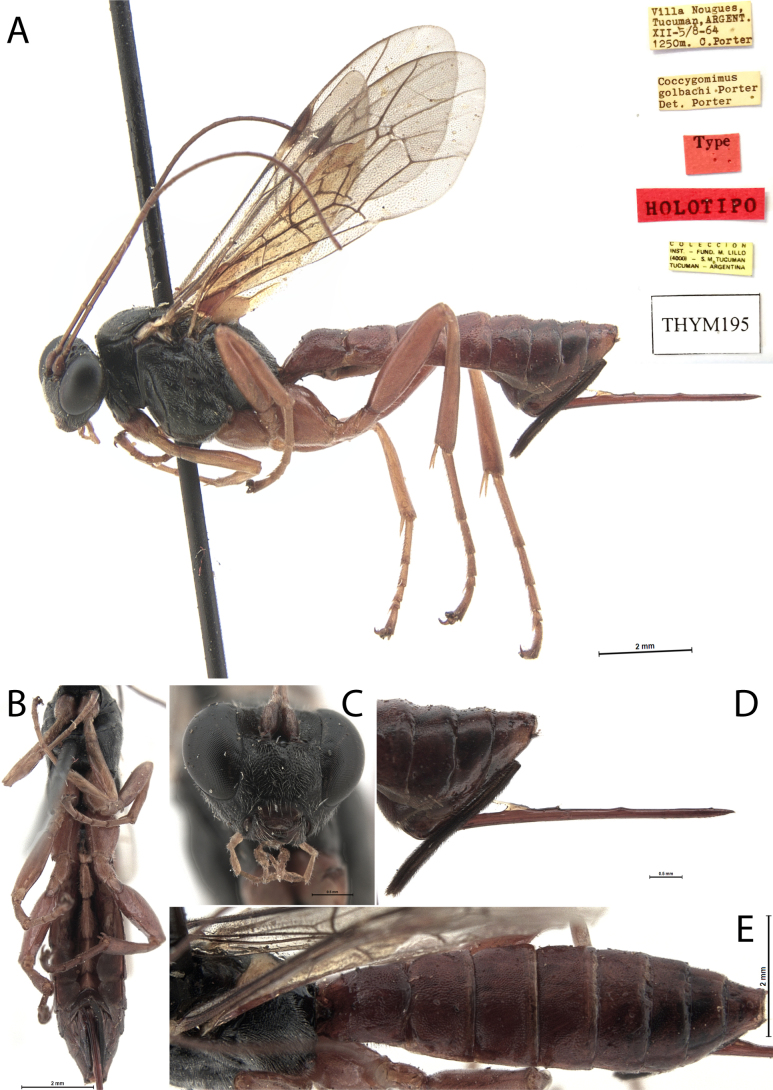
*Pimpla golbachi* (Porter, 1970), female, holotype. **A**. Body, lateral view; **B**. Body, ventral view; **C**. Face, frontal view; **D**. Ovipositor, lateral view; **E**. Metasoma, dorsal view.

**Figure 4.**
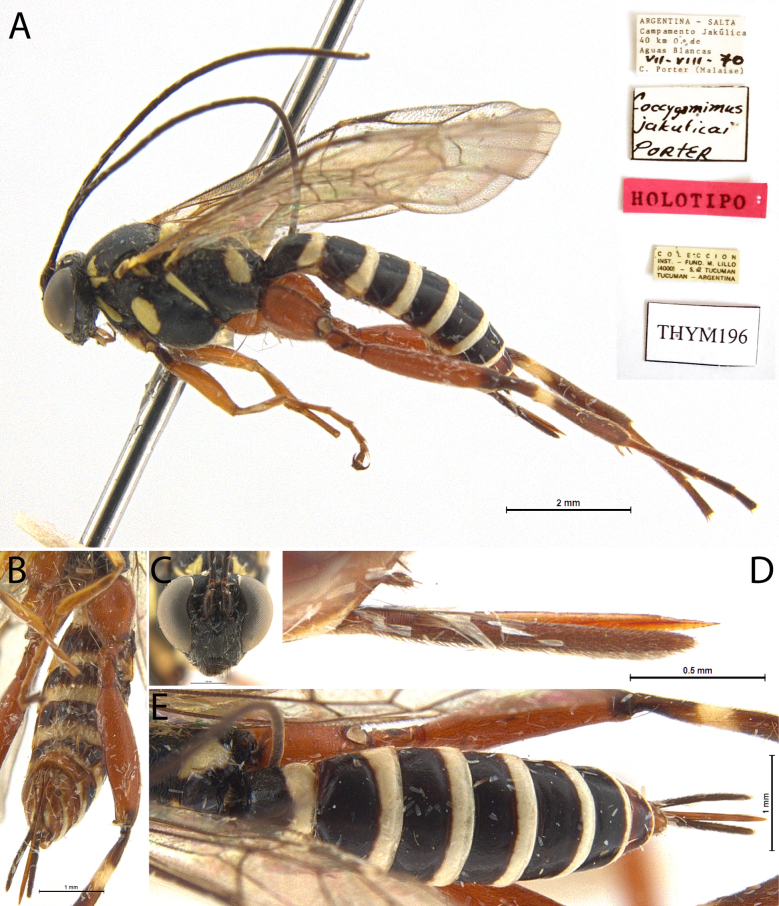
*Pimpla jakulicai* (Porter, 1972), female, holotype. **A**. Body, lateral view; **B**. Metasoma, ventral view; **C**. Face, frontal view; **D**. Ovipositor, lateral view; **E**. Metasoma, dorsal view.

**Figure 5.**
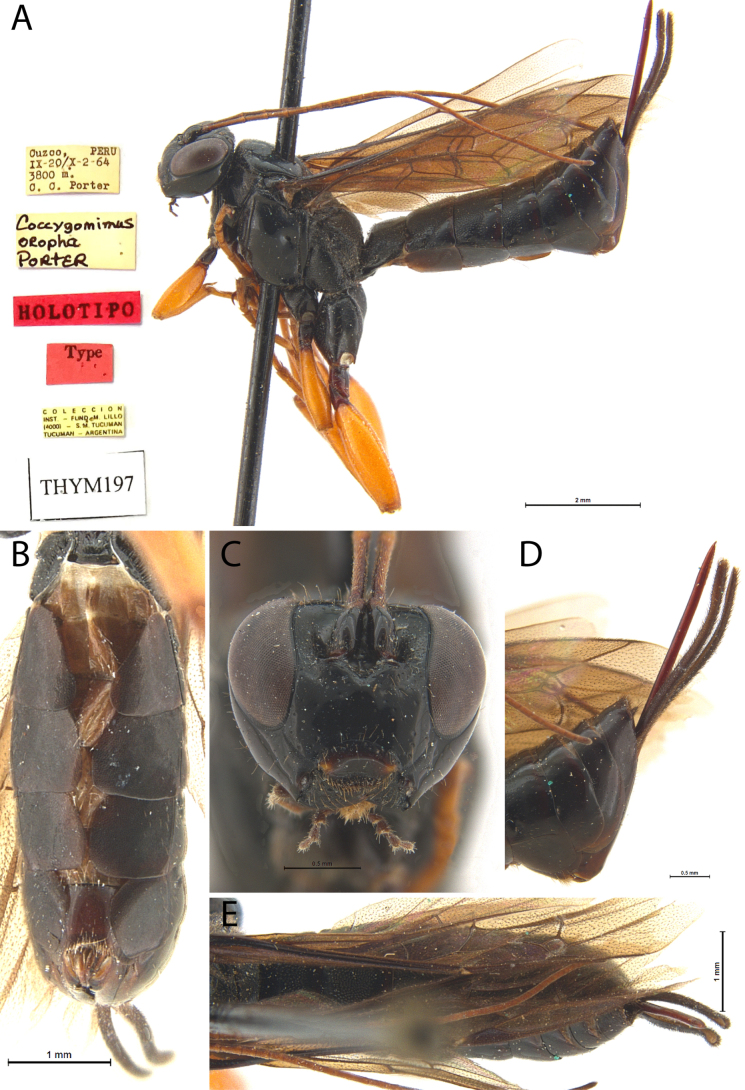
*Pimpla oropha* (Porter, 1970), female, holotype. **A**. Body, lateral view; **B**. Metasoma, ventral view; **C**. Face, frontal view; **D**. Ovipositor, lateral view; **E**. Metasoma, dorsal view.

**Figure 6.**
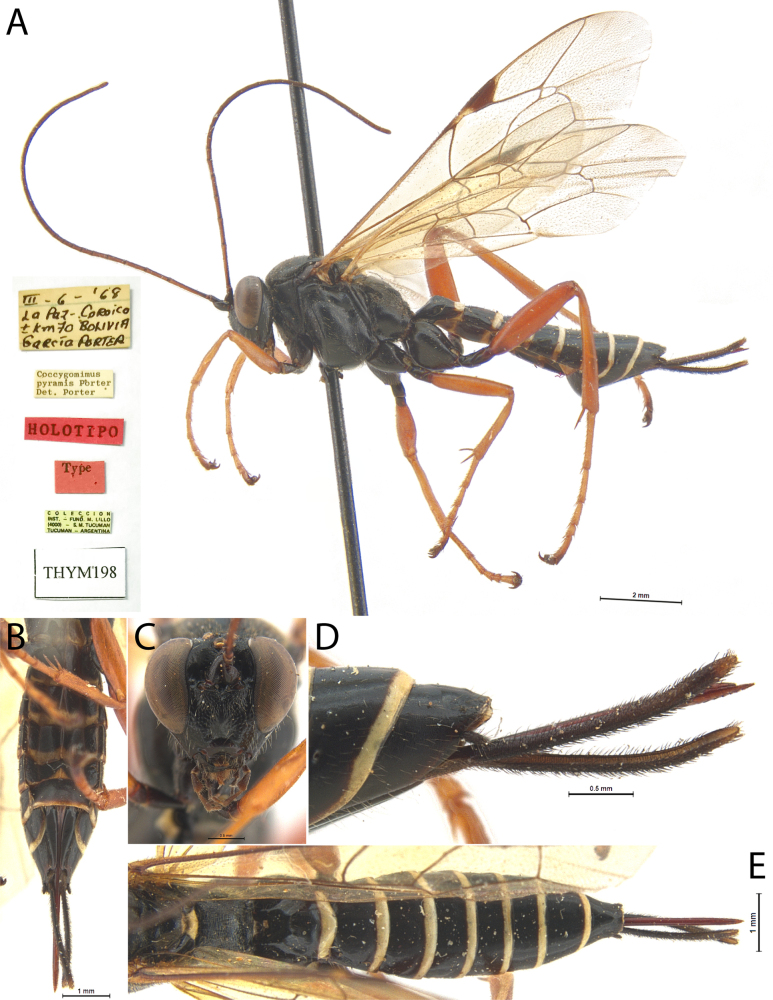
*Pimpla pyramis* (Porter, 1970), female, holotype. **A**. Body, lateral view; **B**. Metasoma, ventral view; **C**. Face, frontal view; **D**. Ovipositor, lateral view; **E**. Metasoma, dorsal view.

**Figure 7.**
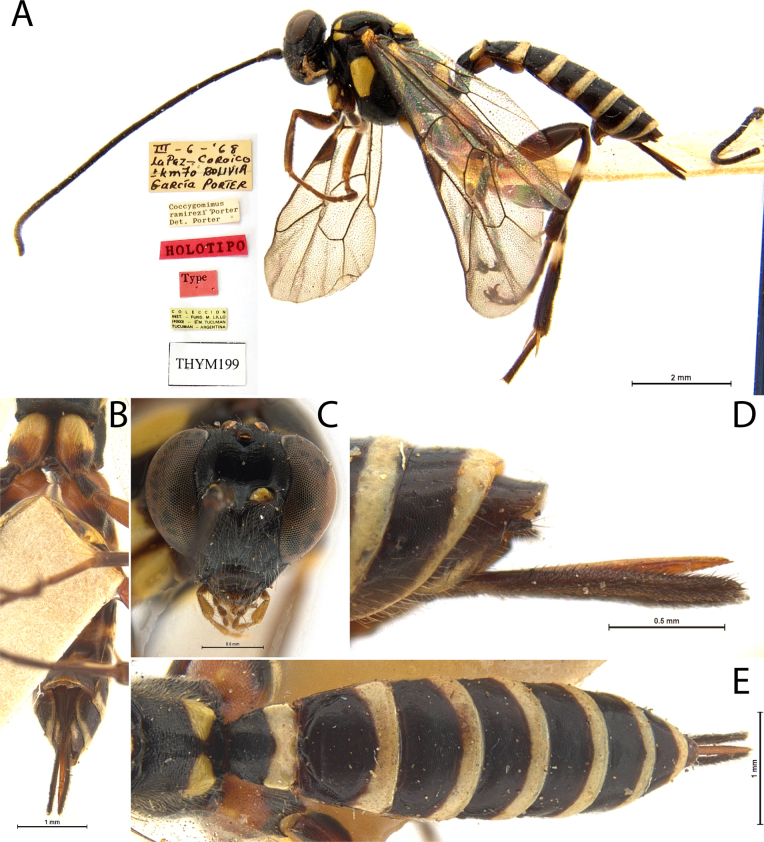
*Pimpla ramirezi* (Porter, 1970), female, holotype. **A**. Body, lateral view; **B**. Metasoma, ventral view; **C**. Face, frontal view; **D**. Ovipositor, lateral view; **E**. Propodeum and metasoma, dorsal view.

**Figure 8.**
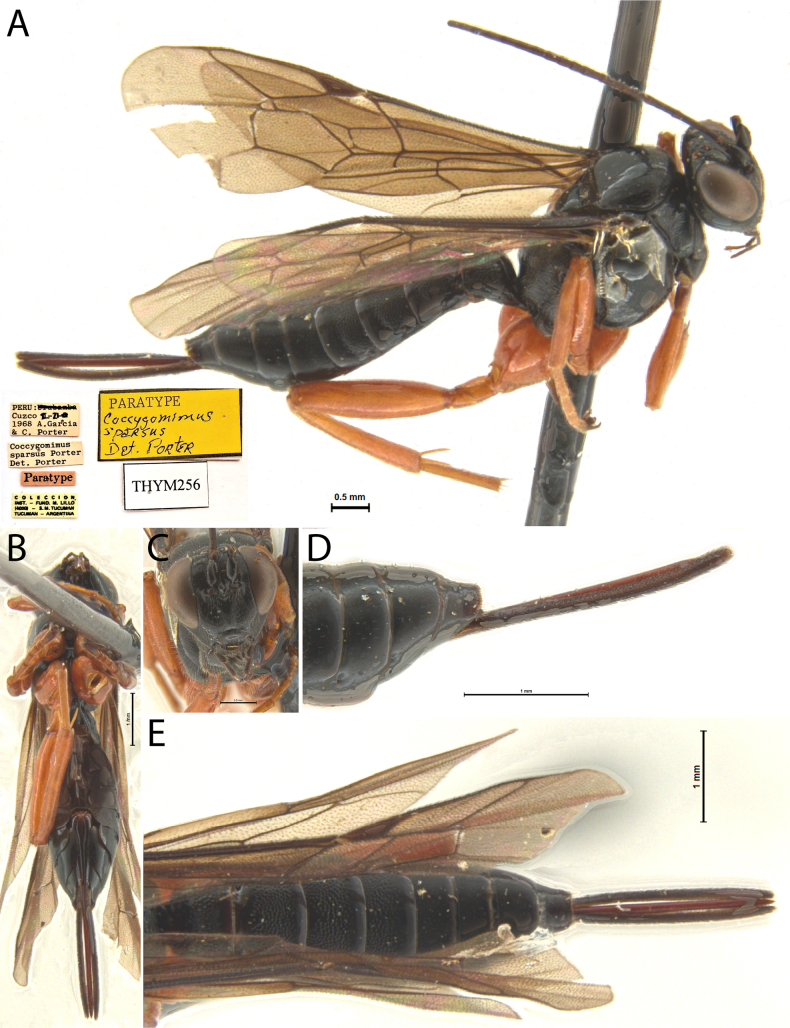
*Pimpla sparsa* (Porter, 1970), female, paratype. **A**. Body, lateral view; **B**. Body, ventral view; **C**. Face, frontal view; **D**. Ovipositor, lateral view; **E**. Metasoma, dorsal view.

This species can be distinguished from the other Neotropical species of *Pimpla* by the combination of the following character states: 1) laterotergite V 1.6 × longer than wide (2.8 × in male) (Fig. 1D); 2) metasoma deep metallic blue to blue-green, usually with a greener apex and faint brownish staining along the apical margins of the tergites (Fig. 1A, C); 3) mesosoma predominantly black, variable metallic reflections, propodeum and metapleuron with metallic sheen (tegula entirely dark) (Fig. 1A); 4) legs predominantly dark to orange-yellow (coxae mostly black, trochanters and trochantelli dark to partly orange, femora orange, and tibiae and tarsi yellow, with distal tarsomeres darker) (Fig. 1A); 5) wings dark with brilliant metallic blue and purple reflections (Fig. 1A); 6) malar space 1.0–1.3 × the basal width of the mandible (Fig. 1B); 7) tergite II smooth and polished, with microreticulation faint and punctation sparse to nearly absent (Fig. 1C); 8) female with ovipositor cylindrical, dorsal valve gently convex on tip; 9) ovipositor sheath about 1.1 × as long as hind tibia (adapted from Porter 1970).

##### Host.

Unknown.

##### Distribution.

Chile and Peru (Yu et al. 2016).

##### Material examined.

Paratype, male. Peru • [Urubamba crossed out] Cuzco, II.7.1968, A. Garcia & C. Porter // Coccygomimus aeolus Porter, det. Porter // Paratype // Coleccion Inst. Fund. M. Lillo (4000) S.M. Tucuman, Tucuman, Argentina // THYM260.

##### Comments.

This species belongs to the *P. albomarginata* species group (*sensu*Porter 1970) (as *Albomarginatus*). According to Porter (1970), the *P. albomarginata* group comprises mostly rare and poorly known species, taxonomically recognized by a combination of morphological characters, including metasomal tergites usually marked with white bands, the female clypeus often deeply emarginate, the laterotergite generally long and narrow, and the apex of the dorsal valve of the ovipositor gently convex and lacking ridges.

#### Pimpla alnorum

Taxon classificationAnimaliaHymenopteraIchneumonidae

(Porter, 1970)

CA7F1A4A-7715-525E-A987-D4A1376E8DC2

Coccygomimus alnorum Porter, 1970: 178. Holotype male, Argentina (EMUS) (Fig. 2).

##### Diagnosis.

This species can be distinguished from the other Neotropical species of *Pimpla* by the combination of the following character states: 1) laterotergite V 1.5 × longer than wide (Fig. 2D); 2) metasoma shining black (Fig. 2C); 3) mesosoma shining black (Fig. 2A); 4) legs mostly orange (coxae black, trochanters and trochantelli blackish to partly pale-marked, hind tibia with a pale premedian annulus and apical infuscation, hind tarsus darkened, especially apically) (Fig. 2A); 5) wings hyaline (Fig. 2A); 6) malar space 0.8–0.9 × the basal width of the mandible (Fig. 2B); 7) tergite II shining, with abundant, coarse, partly confluent punctures (Fig. 2C).

**Female**. Unknown.

##### Host.

Unknown.

##### Distribution.

Argentina (Yu et al. 2016).

##### Material examined.

***Paratype***, male. Argentina • Tucumán RA, Tafí del Valle, La Quebradita, X.10.1968, C. Porter // Coccygomimus alnorum Porter, det. Porter // Paratype // Paratipo // Coleccion Inst. Fund. M. Lillo (4000) S.M. Tucuman, Tucuman, Argentina // THYM1377.

##### Comments.

The female of this species is unknown, and only the male has been described. Porter (1970) considered *P. alnorum* very close to *P. sparsa*, differing only in minor characters, and suggested that it may represent a southern geographic race pending discovery of the female. This species belongs to the *P. aequalis* species group (*sensu*Porter 1970). The *P. aequalis* group is characterized mainly by the laterotergites broad and nearly uniform in width throughout (Porter 1970).

#### Pimpla golbachi

Taxon classificationAnimaliaHymenopteraIchneumonidae

(Porter, 1970)

63F73E29-BD0E-595A-AEEF-4FDCA2EFA986

Ephialtes kreibohmi Blanchard, 1942; *nomen nudum* according to Townes and Townes (1966: 29).
Coccygomimus golbachi Porter, 1970: 153. Holotype female, Argentina (IFML) (Fig. 3).

##### Diagnosis.

**Figure 9.**
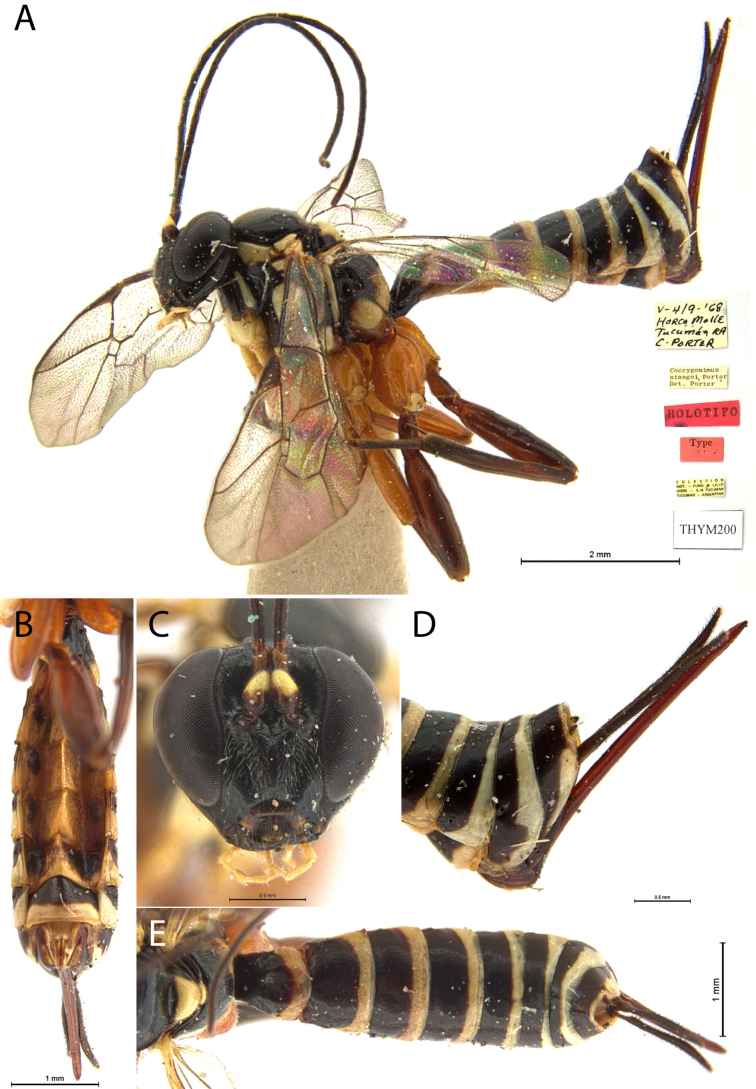
*Pimpla stangei* (Porter, 1970), female, holotype. **A**. Body, lateral view; **B**. Metasoma, ventral view; **C**. Face, frontal view; **D**. Ovipositor, lateral view; **E**. Metasoma, dorsal view.

This species can be distinguished from the other Neotropical species of *Pimpla* by the combination of the following character states: 1) laterotergite V 1.3 × longer than wide (Fig. 3B); 2) metasoma reddish (Fig. 3E); 3) mesosoma black with hind corners of mesopleuron and metapleuron brown (tegula white) (Fig. 3A); 4) legs reddish, except fore coxa often more or less broadly blackish basally, hind tibia sometimes slightly dusky, especially near apex, tarsi usually duller often slightly dusky on apical segment (Fig. 3A); 5) wings hyaline (Fig. 3A); 6) malar space 0.8–1.0 × the basal width of the mandible (0.6–0.9 × in male) (Fig. 3C); 7) tergite II shiny and with almost uniformly distributed large, deep, adjacent to reticulately confluent punctures, except narrowly smooth on apex (Fig. 3E); 8) female with ovipositor cylindrical, dorsal valve with apex without teeth and ventral valve with gently convex teeth on tip (Fig. 3D); 9) ovipositor sheath about 1.45–1.7 × as long as hind tibia (Pádua et al. 2020).

##### Host.

*Pectinophora gossypiella* (Saunders, 1844) (Gelechiidae); *Alabama argillacea* (Hübner, 1818) (Noctuidae); *Colias lesbia* (Fabricius, 1775) (Pieridae); *Diaphania hyalinata* (Linnaeus, 1767) (Pyralidae), and *Rhyacionia buoliana* (Denis & Schiffermüller, 1775) (Tortricidae) (Yu et al. 2016).

##### Distribution.

Argentina, Bolivia, Brazil, Colombia, Paraguay, and Uruguay.

##### Material examined.

Holotype, female. Argentina • Tucuman, Villa Nougues, 1250 m., XII.5–8.1964, C. Porter // Coccygomimus golbachi Porter, det. Porter // Type // Holotipo // Coleccion Inst. Fund. M. Lillo (4000) S.M. Tucuman, Tucuman, Argentina // THYM195.

##### Comments.

This species belongs to the *Pimpla sodalis* species group (*sensu*Porter 1970). The *P. sodalis* group is a small, structurally uniform taxon characterized by a strongly, closely punctate postpetiole and second metasomal tergite, and by the relatively long, narrow laterotergites II–III, contrasting with the much broader and shorter laterotergite IV and following (Porter 1970).

#### Pimpla jakulicai

Taxon classificationAnimaliaHymenopteraIchneumonidae

(Porter, 1972)

9F9EE964-6F37-5D72-9AA5-CF48F4C10EBB

Fig. 4

Coccygomimus jakulicai Porter, 1972: 328. Holotype, female, Argentina (IFML).

##### Diagnosis.

This species can be distinguished from the other Neotropical species of *Pimpla* by the combination of the following character states: 1) laterotergite V 4.0–5.0 × longer than wide (Fig. 4B); 2) metasoma predominantly black and shiny, with pale apical band, posterior tergites partly reddish, especially laterally and toward apex (Fig. 4E); 3) mesosoma predominantly black and shiny, with conspicuous pale yellowish to whitish markings on the pronotum, mesoscutum, tegula, scutellum, mesopleuron, and propodeum (Fig. 4A); 4) fore and mid legs predominantly reddish, with contrasting pale markings and some blackish areas, hind leg darker, with tibia and tarsus mostly blackish and only partly reddish (Fig. 4A); 5) wings hyaline (Fig. 4A); 6) malar space 0.91 × the basal width of the mandible (0.6–0.8 × in male) (Fig. 4C); 7) tergite II shining, with distinct fine microreticulation and sparse, shallow, inconspicuous medium to moderately large punctures (Fig. 4E); 8) female with ovipositor cylindrical-compressed, dorsal valve weakly convex on tip (Fig. 4D); 9) ovipositor sheath about 0.6 × as long as hind tibia.

##### Host.

Unknown.

##### Distribution.

Argentina (Yu et al. 2016).

##### Material examined.

***Holotype***, female. Argentina • Salta, Campamento Jakúlica, 40 km O. de Aguas Blancas, VII–VIII.1970, Malaise [trap], C. Porter // Coccygomimus jakulicai Porter // Holotipo // Coleccion Inst. Fund. M. Lillo (4000) S.M. Tucuman, Tucuman, Argentina // THYM196.

##### Comments.

This species belongs to the *P. albomarginata* species group; see the comments under *Pimpla aeola*.

#### Pimpla oropha

Taxon classificationAnimaliaHymenopteraIchneumonidae

(Porter, 1970)

10B6C22A-87BF-52C7-AC20-7ECCDDF4455A

Fig. 5

Coccygomimus oropha Porter, 1970: 172. Holotype, female, Peru (IFML).

##### Diagnosis.

This species can be distinguished from the other Neotropical species of *Pimpla* by the combination of the following character states: 1) laterotergite V 0.9–1.2 × longer than wide (0.9–1.0 × in male) (Fig. 5B); 2) metasoma shining black (Fig. 5E); 3) mesosoma shining black (Fig. 5A); 4) legs mostly orange (coxae black; trochanters and trochantelli blackish; tarsi slightly dusky) (Fig. 5A); 5) wings moderately to weakly infuscate (without a strongly contrasting apical dark area) (Fig. 5A); 6) malar space 0.9–1.1 × the basal width of the mandible (0.75–0.9 × in male) (Fig. 5C); 7) tergite II shining with large, strong, mostly adjacent to subadjacent punctures and with a wide impunctate apical zone (Fig. 5E); 8) female with ovipositor cylindrical, dorsal valve convex on tip (Fig. 5D); 9) ovipositor sheath about 1.2 × as long as hind tibia.

##### Host.

Unknown.

##### Distribution.

Argentina, Chile, and Peru (Yu et al. 2016).

##### Material examined.

***Holotype***, female. PERU: Cuzco, 3800 m, IX.20–X.2.1964, C.C. Porter // Coccygomimus oropha Porter // Holotipo // Type // Coleccion Inst. Fund. M. Lillo (4000) S.M. Tucuman, Tucuman, Argentina // THYM197.

##### Comments.

This species belongs to the *P. aequalis* species group; see the comments under *Pimpla alnorum*.

#### Pimpla pyramis

Taxon classificationAnimaliaHymenopteraIchneumonidae

(Porter, 1970)

781964DA-D8C3-56A5-ACC3-1B7DB4FE9882

Coccygomimus pyramis Porter, 1970: 122. Holotype, female, Bolivia (IFML) (Fig. 6).

##### Diagnosis.

This species can be distinguished from the other Neotropical species of *Pimpla* by the combination of the following character states: 1) laterotergite V 3.8 × longer than wide (4.8 × in male) (Fig. 6B); 2) metasoma black and shiny, with white apical bands on tergites I–VII, broadest on tergite I and progressively narrower posteriorly (Fig. 6E); 3) mesosoma predominantly black (postscutellum white, scutellum sometimes with a subapical white band, and tegula black, slightly brownish apically) (Fig. 6A); 4) legs reddish except coxae, trochanters and trochantelli darkish (Fig. 6A); 5) wings hyaline weakly yellowish (Fig. 6A); 6) malar space about 0.8–0.9 × the basal width of the mandible (0.5–0.6 × in male) (Fig. 6C); 7) tergite II silky-shining, with fine but distinct microreticulation and sparse, shallow, inconspicuous medium-sized punctures (Fig. 6E); 8) female with ovipositor cylindrical, dorsal valve weakly convex on tip (Fig. 6D); 9) ovipositor sheath about 0.7 × as long as hind tibia.

##### Host.

Unknown.

##### Distribution.

Bolivia, Colombia, and Venezuela (Yu et al. 2016).

##### Material examined.

***Holotype***, female. Bolivia • La Paz, Coroico, +/– km 70, III.6.1968, García & Porter // Coccygomimus pyramis Porter, det. Porter // Holotipo // Type // Coleccion Inst. Fund. M. Lillo (4000) S.M. Tucuman, Tucuman, Argentina // THYM198.

##### Comments.

This species belongs to the *P. albomarginata* species group; see the comments under *Pimpla aeola*.

#### Pimpla ramirezi

Taxon classificationAnimaliaHymenopteraIchneumonidae

(Porter, 1970)

90E64151-1562-5228-A9EF-08FAEB0AEE7B

Coccygomimus ramirezi Porter, 1970: 108. Holotype, female, Bolivia (IFML) (Fig. 7).

##### Diagnosis.

This species can be distinguished from the other Neotropical species of *Pimpla* by the combination of the following character states: 1) laterotergite V 4.8 × longer than wide (4.0 × in male) (Fig. 7B); 2) metasoma black, with broad white apical bands on tergites I–VII, tergite VIII white laterally at apex, brownish staining slight, mostly along margins of white bands (Fig. 7E); 3) mesosoma black, with extensive pale yellow markings on pronotum, mesoscutum, scutellum, mesopleuron, and propodeum (Fig. 7A); 4) legs bicoloured, with fore and mid legs partly reddish to pale-marked, but hind leg predominantly dark, hind tibia black with a short premedian white annulus, and hind tarsus mostly black (Fig. 7A); 5) wings hyaline, slightly dusky toward apex of fore wing (Fig. 7A); 6) malar space 0.9 × the basal width of the mandible (0.6 × in male) (Fig. 7C); 7) tergite II silky-shining, with well-developed, fine microreticulation and widely spaced, superficial and inconspicuous, medium-sized punctures (Fig. 7E); 8) female with ovipositor cylindrical-compressed, dorsal valve weakly convex on tip (Fig. 7D); 9) ovipositor sheath about 0.5 × as long as hind tibia.

##### Host.

Unknown.

##### Distribution.

Argentina and Bolivia (Yu et al. 2016).

##### Material examined.

***Holotype***, female. Bolivia • La Paz, Coroico, +/– km 70, III.6.1968, García & Porter // Coccygomimus ramirezi Porter, det. Porter // Holotipo // Type // Coleccion Inst. Fund. M. Lillo (4000) S.M. Tucuman, Tucuman, Argentina // THYM199.

##### Comments.

This species belongs to the *P. albomarginata* species group; see the comments under *Pimpla aeola*.

#### Pimpla sparsa

Taxon classificationAnimaliaHymenopteraIchneumonidae

(Porter, 1970)

5EFF1F26-054F-5667-B9F4-472B3E1491CF

Fig. 8

Coccygomimus sparsus Porter, 1970: 168. Holotype, female, Peru (EMUS).

##### Diagnosis.

This species can be distinguished from the other Neotropical species of *Pimpla* by the combination of the following character states: 1) laterotergite V 1.5 × longer than wide (Fig. 8B); 2) metasoma shining black (Fig. 8E); 3) mesosoma shining black (Fig. 8A); 4) legs reddish, except fore coxa black (Fig. 8A); 5) wings moderately to rather weakly darkened (Fig. 8A); 6) malar space 1.1–1.2 × the basal width of the mandible (0.9–1.1 × in male) (Fig. 8C); 7) tergite II shiny, with fine microreticulation and abundant coarse punctures, except for a narrow impunctate apical rim (Fig. 8E); 8) female with ovipositor cylindrical-compressed, dorsal valve convex on tip (Fig. 8D); 9) ovipositor sheath about 1.2 × as long as hind tibia.

##### Host.

Unknown.

##### Distribution.

Ecuador and Peru (Yu et al. 2016).

##### Material examined.

***Paratype***, female. PERU: [Urubamba crossed out] Cuzco, II.2(?).1968, A. Garcia & C. Porter // Coccygomimus sparsus Porter, det. Porter // Paratype // Coleccion Inst. Fund. M. Lillo (4000) S.M. Tucuman, Tucuman, Argentina // THYM256.

##### Comments.

This species belongs to the *P. aequalis* species group; see the comments under *Pimpla alnorum*.

#### Pimpla stangei

Taxon classificationAnimaliaHymenopteraIchneumonidae

(Porter, 1970)

ED609619-CF08-5357-B987-E141882898E6

Coccygomimus stangei Porter, 1970: 99. Holotype, female, Argentina (IFML) (Fig. 9).

##### Diagnosis.

This species can be distinguished from the other Neotropical species of *Pimpla* by the combination of the following character states: 1) laterotergite V 4.6–4.8 × longer than wide (3.6 × in male) (Fig. 9B); 2) metasoma black, sometimes variably pale brown stained white apical bands on all tergites (Fig. 9E); 3) mesosoma predominantly black and shiny, with extensive white to pale yellow markings on the pronotum, tegula, scutellum, postscutellum, mesopleuron, metapleuron, and propodeum (metapleuron partly orange-brown to black, with large pale blotches) (Fig. 9A); 4) legs predominantly pale orange to reddish, with fore leg mostly pale, mid leg partly darkened apically, and hind leg distinctly bicoloured to mostly dark, hind femur black with red-brown staining, hind tibia and tarsus blackish to black (Fig. 9A); 5) wings hyaline, weakly infuscate apically (Fig. 9A); 6) malar space 0.6–0.8 × the basal width of the mandible (0.55–0.65 × in male) (Fig. 9C); 7) tergite II moderately shiny, with distinct microreticulation and moderately sparse, shallow, irregularly spaced punctures (Fig. 9E); 8) female with ovipositor cylindrical, dorsal valve weakly convex on tip (Fig. 9D); 9) ovipositor sheath about 0.95 × as long as hind tibia.

##### Host.

Unknown.

##### Distribution.

Argentina and Peru (Yu et al. 2016).

##### Material examined.

***Holotype***, female. Argentina • Tucumán RA, Horco Molle, V.4–9.1968, C. Porter // Coccygomimus stangei, det. Porter // Holotipo // Type // Coleccion Inst. Fund. M. Lillo (4000) S.M. Tucuman, Tucuman, Argentina // THYM200.

##### Comments.

This species belongs to the *P. albomarginata* species group; see the comments under *Pimpla aeola*.

#### Pimpla tafiae

Taxon classificationAnimaliaHymenopteraIchneumonidae

(Porter, 1970)

B15A20A1-DC16-5803-A075-722CB9BEC08A

**Figure 10.**
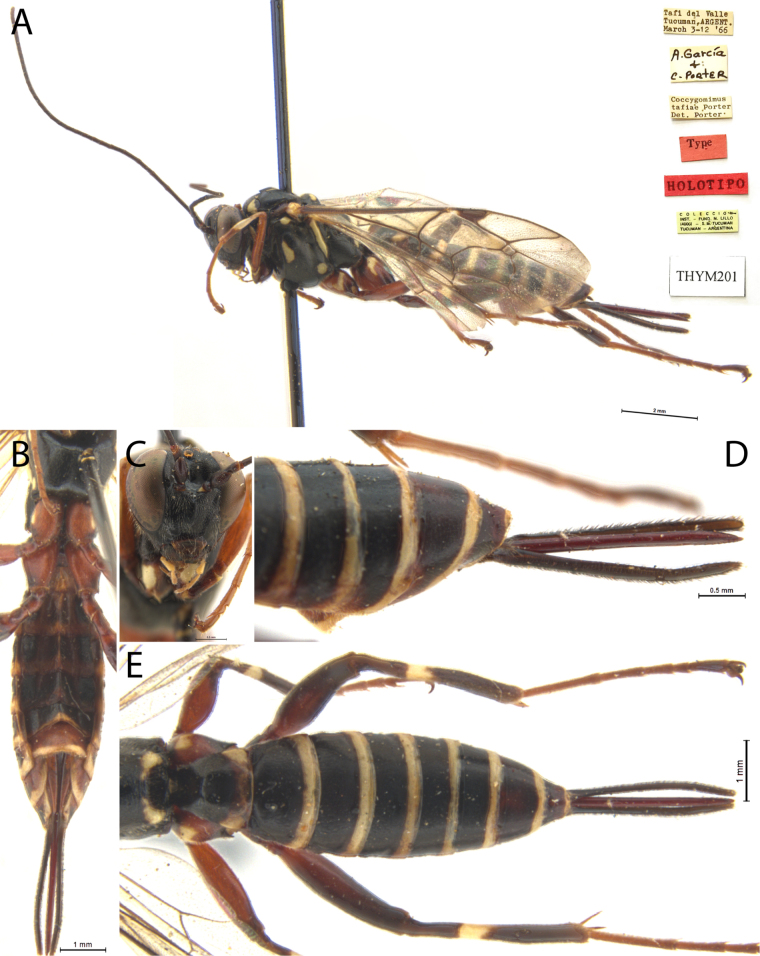
*Pimpla tafiae* (Porter, 1970), female, holotype. **A**. Body, lateral view; **B**. Metasoma, ventral view; **C**. Face, frontal view; **D**. Ovipositor, lateral view; **E**. Metasoma, dorsal view.

Coccygomimus tafiae Porter, 1970: 112. Holotype, female, Argentina (IFML) (Fig. 10).

##### Diagnosis.

This species can be distinguished from the other Neotropical species of *Pimpla* by the combination of the following character states: 1) laterotergite V 4.2 × longer than wide (5.0 × in male) (Fig. 10B); 2) metasoma black, sometimes variably pale brown stained white apical bands on all tergites (Fig. 10E); 3) mesosoma black, with extensive white markings on the pronotum, mesoscutum, tegula, scutellum, subalarum, mesopleuron, and propodeum (Fig. 10A); 4) legs distinctly bicoloured (coxae variously black, red, and white-marked, fore femur red, mid femur red with dusky apex, hind femur predominantly black with reddish markings, fore tibia white-striped, mid and hind tibiae blackish with a narrow premedian white annulus, hind tarsus blackish to black) (Fig. 10A); 5) wings hyaline, faintly brownish apically (Fig. 10A); 6) malar space about 0.7–0.8 × the basal width of the mandible (0.5–0.65 × in male) (Fig. 10C); 7) tergite II slightly dull-shining, with strong fine microreticulation and numerous shallow, irregularly spaced punctures, becoming weaker centrally and apically (Fig. 10E); 8) female with ovipositor cylindrical, dorsal valve weakly convex on tip (Fig. 10D); 9) ovipositor sheath about 0.9 × as long as hind tibia.

##### Host.

*Locha* sp. (Geometridae) (Yu et al. 2016).

##### Distribution.

Argentina and Ecuador (Yu et al. 2016).

##### Material examined.

***Holotype***, female. Argentina • Tucuman, Tafí del Valle, III.3–12.1966 // A. García & C. Porter // Coccygomimus tafiae Porter, det. Porter // Type // Holotipo // Coleccion Inst. Fund. M. Lillo (4000) S.M. Tucuman, Tucuman, Argentina // THYM201.

##### Comments.

This species belongs to the *P. albomarginata* species group; see the comments under *Pimpla aeola*.

#### Pimpla tarapacae

Taxon classificationAnimaliaHymenopteraIchneumonidae

(Porter, 1970)

C7645312-F219-5799-8C56-9409EF7440DB

**Figure 11.**
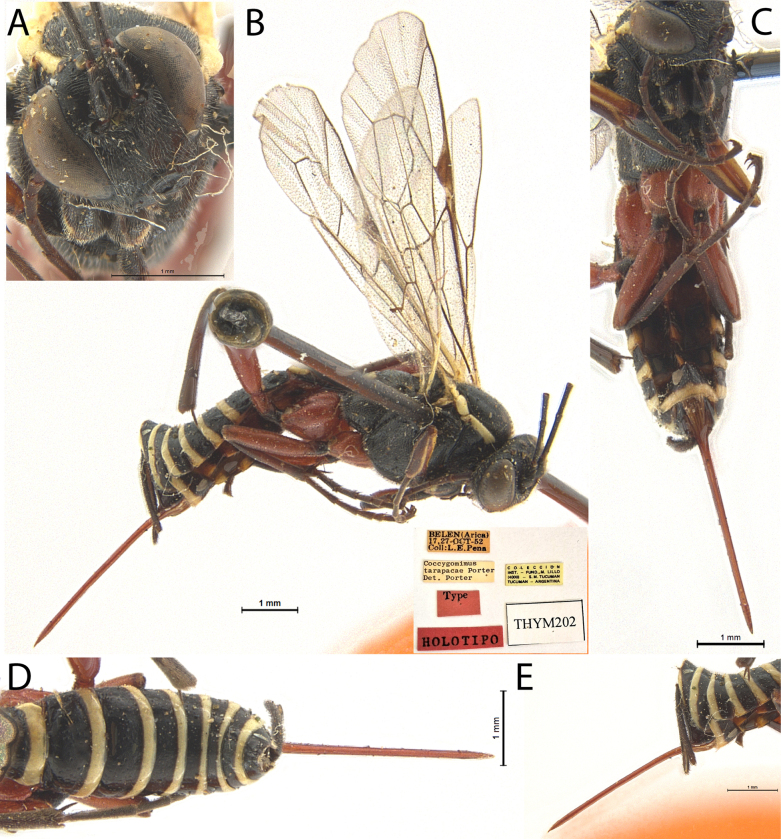
*Pimpla tarapacae* (Porter, 1970), female, holotype. **A**. Face, frontal view; **B**. Body, lateral view; **C**. Body, ventral view; **D**. Metasoma, dorsal view; **E**. Ovipositor, lateral view.

Coccygomimus tarapacae Porter, 1970: 120. Holotype, female, Chile (IFML) (Fig. 11).

##### Diagnosis.

This species can be distinguished from the other Neotropical species of *Pimpla* by the combination of the following character states: 1) laterotergite V 3.8 × longer than wide; 2) metasoma black, sometimes variably pale brown stained white apical bands on all tergites; 3) mesosoma black and shiny, with white markings on the dorsal pronotum, tegula, postscutellum, and upper hind corners of the propodeum (Fig. 11B); 4) legs bicoloured (fore and mid legs mostly red to blackish with limited white markings, hind leg darker, hind femur blackish with reddish markings, hind tibia black, and hind tarsus mostly black); 5) wings hyaline; 6) malar space 0.93 × the basal width of the mandible; 7) tergite II shiny, with distinct microreticulation and irregularly scattered medium to large punctures, these mostly widely spaced; 8) female with ovipositor cylindrical, dorsal valve weakly convex on tip; 9) ovipositor sheath about 0.9 × as long as hind tibia.

**Male**. Unknown.

##### Host.

Unknown.

##### Distribution.

Chile (Yu et al. 2016; Araujo et al. 2025).

##### Material examined.

***Holotype***, female. Chile • Arica [Región de Arica y Parinacota], Belen, 17–27.X.1952, L.E. Pena coll. // Coccygomimus tarapacae Porter, det. Porter // Type // Holotipo // Coleccion Inst. Fund. M. Lillo (4000) S.M. Tucuman, Tucuman, Argentina // THYM202.

##### Comments.

This species belongs to the *P. albomarginata* species group; see the comments under *Pimpla aeola*.

## Discussion

Currently, 16 species of *Pimpla* are recorded for Argentina. According to the species-group classification proposed by Porter (1970), these species are distributed among six main groups. The Aztecus Group is represented by *Pimpla sumichrasti*. The Caeruleus Group includes *P. caerulea*, *P. flavipennis*, and *P. tomyris*. The Croceiventris Group is represented by *P. croceiventris*. The Albomarginatus Group comprises *P. ramirezi*, *P. stangei*, *P. tafiae*, and *P. jakulicai*. The Sodalis Group includes *P. cyanipennis*, *P. golbachi*, and *P. semirufa*. Finally, the Aequalis Group includes *P. alnorum*, known from the male, *P. oropha*, *P. patirrufa* (= rufipes), and *P. fuscipes*. Thus, the Argentine fauna of *Pimpla* comprises representatives of several morphological species groups recognized by Porter, reflecting a relevant taxonomic diversity within Pimplinae in the region.

According to Berta (2013), Dr Charles Porter deposited in the entomological collection of the Fundación Miguel Lillo (IFML) some of the material from his collecting trips, not only in Argentina but in several countries. He identified and classified approximately 14,000 specimens, including some 152 species from Tucumán alone.

## Supplementary Material

XML Treatment for Pimpla

XML Treatment for Pimpla aeola

XML Treatment for Pimpla alnorum

XML Treatment for Pimpla golbachi

XML Treatment for Pimpla jakulicai

XML Treatment for Pimpla oropha

XML Treatment for Pimpla pyramis

XML Treatment for Pimpla ramirezi

XML Treatment for Pimpla sparsa

XML Treatment for Pimpla stangei

XML Treatment for Pimpla tafiae

XML Treatment for Pimpla tarapacae

## Additional information

### Artificial Intelligence (AI) use

The authors accept full responsibility for the content of the manuscript, including the disclosure of any use of AI.

No AI tools were used in the preparation of this manuscript.

### Author ORCIDs

D.G. Pádua https://orcid.org/0000-0001-5061-2978

E.C. Pérez https://orcid.org/0000-0001-7407-1563

R.O. Araujo https://orcid.org/0000-0002-9438-3238

### Data availability

All of the data that support the findings of this study are available in the main text.

## References

[B1] Araujo RO, Fernandes DRR, Dal Pos D, Di Giovanni F, Moreira-Muñoz A, Pádua DG (2025) Unveiling the secrets of South American Darwin wasps, part I: a comprehensive checklist of the Chilean Ichneumonidae (Hymenoptera: Ichneumonoidea). Zootaxa 5631(1): 1–51. 10.11646/zootaxa.5631.1.1 41119227

[B2] Berta DC (2013) Dr. Charles Porter (1940–2012). Acta Zoológica Lilloana 57(1): 138.

[B3] Blanchard EE (1942) Division de zoologia agrícola. Boletín Informativo Direccion Sanidad Vegetal 6(21): 8–9.

[B4] Brèthes J (1916) Hyménoptères parasites de l’Amérique méridionale. Anales del Museo Nacional de Historia Natural de Buenos Aires 27: 401–430.

[B5] Brèthes J (1927) Hyménoptères sud-américains du Deutsches Entomologisches Institut: Terebrantia. Entomologische Mitteilungen 16: 319–335.

[B6] Broad GR, Shaw MR, Fitton MG (2018) Ichneumonid Wasps (Hymenoptera: Ichneumonoidea): Their Classification and Biology. RES Handbooks for the Identification of British Insects 7(12). Field Studies Council, Shrewsbury, 418 pp. 10.1079/9781800625471.0000

[B7] Cameron P (1900) HymenopteraOrientalia, or contributions to the knowledge of the Hymenoptera of the Oriental zoological region. Part IX. The Hymenoptera of the Khasia Hills. Part II. Section I. Memoirs and Proceedings of the Manchester Literary and Philosophical Society 44: 1–114. 10.5962/bhl.part.9307

[B8] Cameron P (1905) On the phytophagous and parasitic Hymenoptera collected by Mr. E. Ernest Green in Ceylon. Spolia Zeylanica 3: 67–143.

[B9] Díaz FA (2000) The Venezuelan species of *Pimpla* (Hymenoptera: Ichneumonidae). Journal of Hymenoptera Research 9(2): 246–253.

[B10] Enderlein G (1919) Beiträge zur Kenntnis aussereuropäischer Ichneumoniden. IV. Einige neue Pimpliden. Sitzungsberichte der Gesellschaft Naturforschender Freunde zu Berlin 1919: 146–153.

[B11] Fabricius JC (1804) Systema Piezatorum: secundum ordines, genera, species, adiectis synonymis, locis, observationibus, descriptionibus. Carolum Reichard, Brunsvigae, 469 pp. 10.5962/bhl.title.10490

[B12] Gauld ID (1991) The Ichneumonidae of Costa Rica I. Memoirs of the American Entomological Institute 47: 1–589.

[B13] Gauld ID, Gómez JAU, Hanson PS (1998) Guía de los Pimplinae de Costa Rica (Hymenoptera: Ichneumonidae). Revista de Biología Tropical 46: 1–189.

[B14] Gauld ID, Menjivar R, Monro A, Gonzalez MO (2002) Guía para la identificación de los Pimplinae de cafetales bajo sombra de El Salvador (Hymenoptera: Ichneumonidae). Natural History Museum, London, 1–76.

[B15] International Commission on Zoological Nomenclature [ICZN] (1945) Opinion 159. On the status of the names *Ephialtes* Schrank, 1802, *Ichneumon* Linnaeus, 1758, *Pimpla* Fabricius (1804–1805), and *Ephialtes* Gravenhorst, 1829 (Class Insecta, Order Hymenoptera). Opinions and Declarations Rendered by the International Commission on Zoological Nomenclature 2(29): 275–290. 10.5962/p.46726

[B16] Khalaim AI, Ruíz-Cancino E (2021) Darwin wasps of the subfamily Pimplinae (Hymenoptera: Ichneumonidae) of Mexico: genera *Apechthis* Förster, *Itoplectis* Förster and *Pimpla* Fabricius. Zootaxa 5071(4): 451–491. 10.11646/zootaxa.5071.4.1 35390898

[B17] Mason WRM (1975) A new genus of Pimplini from Jamaica (Hymenoptera: Ichneumonidae). Proceedings of the Entomological Society of Washington 77: 225–227.

[B18] Morley C (1913) The Fauna of British India Including Ceylon and Burma. Hymenoptera. Vol. 3. Ichneumonidae. British Museum, London, 531 pp.

[B19] Noskiewicz J, Chudoba S (1951) Les suppléments à la faune des Ichneumonides de la Pologne. Polskie Pismo Entomologiczne 21: 30–60.

[B20] Pádua DG, Araujo RO, Mazariegos LA (2019) *Pimpla* Fabricius (Hymenoptera: Ichneumonidae: Pimplinae) from Colombia. Zootaxa 4683(3): 439–446. 10.11646/zootaxa.4683.3.8 31715922

[B21] Pádua DG, Fernandes DRR, Sääksjärvi IE (2020) *Pimpla* Fabricius, 1804 (Ichneumonidae, Pimplinae) from Uruguay: a replacement name, new records, and an identification key to the species. ZooKeys 1007: 23–47. 10.3897/zookeys.1007.56328 PMC7788073 33505181

[B22] Porter CC (1970) A revision of the South American species of *Coccygomimus* (Hymenoptera, Ichneumonidae). Studia Entomologica 13: 1–192.

[B23] Porter CC (1972) A new Argentine species of *Coccygomimus* (Hymenoptera, Ichneumonidae). Psyche 79(4): 328–334. 10.1155/1972/68204

[B24] Saussure H (1892) Hyménoptères. In: Grandidier A (Ed.) Histoire Physique, Naturelle et Politique de Madagascar, 20. Paris, 590 pp.

[B25] Seyrig A (1932) Les Ichneumonides de Madagascar. I. IchneumonidaePimplinae. Mémoires de l’Académie Malgache 11: 1–183.

[B26] Townes HK, Townes M (1966) A catalogue and reclassification of the Neotropic Ichneumonidae. Memoirs of the American Entomological Institute 8: 1–367.

[B27] Viereck HL (1914) Type species of the genera of Ichneumon flies. Bulletin of the United States National Museum 83: 1–186. 10.5479/si.03629236.83.1

[B28] Villanueva-Bonilla GA, Pádua DG, Sobczak JF (2021) New records of *Pimpla* Fabricius, 1804 (Hymenoptera, Ichneumonidae, Pimplinae) from Brazilian northeast. Check List 17(1): 159–165. 10.15560/17.1.159

[B29] Watanabe K, Matsumoto R (2019) Review of the Genus *Pimpla* Fabricius, 1804 (Hymenoptera: Ichneumonidae, Pimplinae) from Japan. Japanese Journal of Systematic Entomology 25(2): 217–224.

[B30] Yu DS, van Achterberg C, Horstmann K (2016) World Ichneumonoidea 2015: Taxonomy, Biology, Morphology and Distribution. Taxapad 2016. Nepean, Ontario [database on flash drive].

